# An evaluation of the construct of emotional sensitivity from the perspective of emotionally sensitive people

**DOI:** 10.1186/s40479-018-0091-y

**Published:** 2018-08-23

**Authors:** Kiana Wall, Allison Kalpakci, Karyn Hall, Nicholas Crist, Carla Sharp

**Affiliations:** 1 Department of Psychology, University of Houston, 4800 Calhoun Rd, Houston, TX 77004 USA; 2 Dialectical Behavior Therapy Center of Houston, 920 Frostwood Dr, Houston, TX 77024 USA

**Keywords:** Emotional sensitivity, Borderline personality disorder, Qualitative, Patient perspective

## Abstract

**Background:**

Emotional sensitivity is a construct found in major developmental models of borderline personality disorder. However, the construct remains nebulous. The patient perspective is crucially important in helping to define and conceptualize any psychological construct – especially one that plays such a large role in the developmental theories of a given disorder. The aim of the current study was to explore the meaning of emotional sensitivity from the perspective of those who identify as being emotionally sensitive.

**Methods:**

Participants were from a community sample of adults (*M*_*age*_ = 32.05, range: 21–59) who responded to an advertisement for a study of emotional sensitivity. Participants completed surveys related to personality pathology and a semi-structured interview about emotional sensitivity. Emotional sensitivity interviews were independently coded by two research assistants trained in qualitative analyses for content and process. Coders were blind to the personality pathology status of participants.

**Results:**

Regardless of level of personality pathology, qualitative results of the emotional sensitivity interview largely suggest that emotional sensitivity is a heightened emotional reactivity to stimuli, including the emotions of other individuals, or a tendency to have emotional reactions to even low impact stimuli. However, emotional sensitivity was regarded predominantly as a negative trait (i.e. burden) only by those who have high levels of borderline personality pathology.

**Conclusions:**

The implications of these results for the conceptualization and utility of emotional sensitivity in borderline personality disorder are discussed.

## Background

The phrase “emotionally sensitive” and other variants are utilized frequently in colloquial settings to identify individuals whom others may describe as sensitive, dramatic and over-reactive. Clinically, emotionally sensitive people have been described as “those who experience intense emotions more frequently and for longer periods of time” [1]. Emotional sensitivity (ES) also serves as a construct within multiple developmental models of borderline personality disorder [2, 3] and is implicated in others [4, 5].

Marsha Linehan’s biosocial theory of borderline personality disorder (BPD) posits that ES is one component of a biologically-based vulnerable temperament which interacts with an invalidating emotional environment, contributing to the development of BPD [2, 3]. Individuals with BPD are thought to be emotionally sensitive from birth, resulting in a higher likelihood of experiencing negative emotions in more situations compared to others. This frequent negative affect makes it more difficult to learn appropriate emotion regulation strategies and increases the likelihood of an individual using a maladaptive strategy [6]. In this way, ES can be viewed as one piece of a dynamic emotion dysregulation process [7]. This process, outlined by Carpenter and Trull, begins with an individuals’ underlying ES. ES may be defined as heightened emotional reactivity or a tendency to respond emotionally to even low-intensity environmental stimuli [6, 8]. As a consequence of ES, individuals with BPD frequently experience more intense, more negative and more unstable emotions than individuals without BPD when encountering a variety of environmental stimuli [6]. Finally, individuals with BPD are often unable to adequately regulate these strong, negative emotions [6]. This may be because their frequent, intense experiences of negative affect make it challenging to learn the skills fundamental to regulating ones emotions [3]. Often, these individuals go on to develop maladaptive, behavioral coping strategies instead [5]. Negative consequences related to experiencing unregulated, intense negative affect can heighten ones ES, resulting in a positive feedback loop.

Despite its inclusion in the biosocial theory of BPD and models of emotion dysregulation, ES remains vaguely defined and has no standardized or universally accepted measure or measurement technique [9]. This is part of a greater problem affecting emotion regulation literature in general, which is wrought with unclear and blurred working definitions of the various constructs it contains [10]. For example, measurement techniques of ES include self-report of ES, intensity and persistence [11, 12], assessment of emotional attentional bias [13], face-morph tasks of emotion perception and identification accuracy [14], and functional magnetic resonance imaging (fMRI) investigations of emotional scenes and facial stimuli (for a review: [15]). Furthermore, ES may be assessed indirectly as hypervigilance [16] to emotional words in a Stroop task or as a bias for identifying negative emotions in others [17].

This work has primarily aimed to identify differences in ES between borderline and healthy populations and due to differences in measurement and design, literature is mixed on whether individuals with BPD show heightened or reduced ES. For example, Jovev and colleagues found that youth with elevated borderline features did not demonstrate earlier, accurate identification of emotions when compared to community participants on a face morph task [14]. In a later study, those same youth did demonstrate an attentional bias for emotion, however this was only for fear [13]. A review of empirical literature highlights discrepant findings for emotion recognition [17]. BPD patients have, in some studies, demonstrated less accurate emotion recognition, lower detection thresholds for emotions and over-reporting of negative emotions, but in others, have not differed from healthy controls on similar indices. Similar discrepant findings have been demonstrated physiologically, as measured by fMRI, in that although literature may agree upon where ES-related activity is taking place in the brain, it is unclear whether increased or decreased activity is taking place [15].

However, it also remains unclear what is really meant with the term “emotional sensitivity”. Based on the above experimental approaches, the phrase “emotional sensitivity” may be used to refer to emotional reactivity speed or likelihood, emotion recognition or identification accuracy, bias for experiencing affect as negative or general emotional hypervigilance. While all may potentially measure ES or components of ES, lacking in the literature to date is the perspective of individuals who regard themselves as emotionally sensitive. The patient perspective is crucially important in helping to define and conceptualize any psychological construct – especially one that plays such a large role in the developmental theories of a given disorder. Without it, any attempt to operationalize or measure the construct is based solely on theory and lacks the valuable insight of subjective experience. In the case of ES, the patient perspective may illuminate whether individuals with and without borderline features differ and which measurement techniques or conceptualizations capture the experiential reality of the construct.

Against this background, the aim of the current study was to use qualitative methodology to explore the meaning and experience of ES among those who self-identify as emotionally sensitive. Given the centrality of ES in models of BPD, we also included two measures of borderline pathology in the study to explore group differences between those with high and low levels of borderline features. Although prior work has demonstrated mixed findings in this regard, we expected that content and process differences would emerge between those with high and low levels of borderline traits, when speaking about ES.

## Methods

### Participants

Participants were recruited via two sources. The first source was an online posting to a blog about ES, written by the director of a Dialectical Behavior Therapy (DBT; [3]) clinic. DBT is a treatment originally developed for individuals with chronically suicidal behavior and BPD. The posting read: “I am going to be starting a research study soon and I’d like to interview a few people about what emotional sensitivity means to you. If you are interested in being interviewed, please email me your contact information.” The second source was an online community posting, seeking participants who identified as being emotionally sensitive. The inclusion criterion for the study was that participant age be 18 years or greater. Participants responding to either advertisement completed informed consent, a demographics survey and two measures of personality pathology via email. Each participant was then contacted via telephone, to complete a semi-structured interview about ES. All study procedures were approved by local ethics boards.

### Measures

#### The McLean Screening Instrument for Borderline Personality Disorder (MSI-BPD [18])

The MSI-BPD is a ten-item instrument intended to screen for BPD. Each item is presented in the form of a question (i.e. “have you frequently felt unreal or as if things around you were unreal?”) and requires a “yes” or “no” response, where “yes” signifies the presence of BPD symptoms. Responses are summed (yes = 1, no = 0) for a total score out of ten. In the current study, an elevated score of five was utilized to distinguish a participant as a member of the group with high borderline features.

#### The Personality Assessment Inventory – Borderline Features Scale (PAI-BOR [19])

The PAI-BOR is a twenty-four item self-report, dimensional measure of borderline personality disorder. The PAI-BOR contains four subscales with six items each, organized around the four symptom areas of BPD: affective instability, identity problems, negative relationships and self-harm. Reponses are made on a four-point scale (0 = false, 1 = slightly true, 2 = mainly true, and 3 = very true) and summed for a continuous total score, with higher scores indicating greater borderline features. The PAI manual [19] suggests a t-score of 60 to 69 on the PAI-BOR scale signifies moderate elevations in moodiness, sensitivity and identity uncertainty. Individuals with t-scores above 70 may be angry, impulsive, feel misunderstood and suspicious. T-scores above 90 are associated with borderline personality functioning. Excellent psychometric properties have been established for this measure, with the present study reporting α = .94.

#### The Emotional Sensitivity Interview

The ES interview aimed at further clarifying the meaning and experience of ES from a subjective point of view. Each question was left open-ended with minimal interviewer prompting. The questions were as follows: 1) What does emotional sensitivity mean to you? 2) Do you consider yourself an emotionally sensitive person? Have you always been an emotionally sensitive person? 3) How do you think your family environment reacted to your emotional sensitivity when you were growing up? How do your family/friends react to your emotional sensitivity now? 4) When you struggled emotionally as a child, how would your family react? 5) Do you think your emotional sensitivity has caused problems for you? 6) How has emotional sensitivity been beneficial to you? Have you ever wanted to change it?

##### Procedures and qualitative data analytic strategy

Participants were assigned to one of two groups, based on whether they displayed high or low levels of borderline traits on the MSI-BPD ([16]; score > 5) and/or the PAI-BOR ([17]; t score > 70). Participants were initially assigned to either group based on the PAI-BOR. Eight participants had a t-score of 70 or above and were assigned to the high borderline features group. Eleven participants had a PAI-BOR t-score of less than 70 and were assigned to the low borderline features group. One participant did not have an available overall PAI-BOR t-score due to incomplete responding. This participant was assigned to the high borderline group, given an MSI total score of 6. Two individuals with MSI scores of only 2 and 3 were retained in the high borderline group given their elevated t-scores of 82 and 70, respectively, on the PAI-BOR. A final participant with a PAI-BOR t-score of only 60 was re-assigned to the high borderline group, given an MSI-BPD of 5. The mean age of the groups, displaying high and low levels of borderline traits respectively, was 33.8 (SD = 10.9) and 30.3 (SD = 9.9). Independent sample t-tests revealed that this difference was not statistically significant (Table 2). Each group consisted of two male and eight female participants.

Emotional sensitivity interviews were audio-recorded and transcribed. They were then independently analyzed by two research assistants, both blind to level of personality pathology, for qualitative content and processes using a modified contextualized content analysis approach [20]. Qualitative analysis of each interview took place in two steps. First, major themes, quotes and examples were pulled for each question. Second, notes were made regarding “how” responses were given including coherence of response, affect during the interview and so on. Notes for all interviews were then combined into one document for direct comparison and level of borderline features was revealed. Each coder summarized major themes and process notes for each group. The independent coders then met and independent analyses were combined, and consensus was reached for final thematic and process results. To ensure validity of the qualitative results of the current study, efforts were made to follow best practices in qualitative research. These include using low inference descriptors (i.e. quotes) in publication, corroboration of conclusions through the use of more than one source of data, using more than one qualitative rater, using both quantitative and qualitative methodology and finally, self-awareness or self-reflection by investigators in the discussion of the current study’s limitations [21, 22].

## Results

### Sample characteristics

Sample characteristics for each group are presented in Table 1. Independent sample t-tests revealed significant mean differences on MSI-BPD total scores and PAI-BOR subscales and total scores between the group above clinical cut-off for BPD and the group below clinical cut-off (Table 2).

**Table 1:** Sample Descriptive Statistics

	Low borderline trait group			High borderline trait group		
	N	Mean	SD	N	Mean	SD
Age in years	10	30.30	9.93	10	33.80	10.90
MSI-BPD Total Score	6	1.67	1.97	10	6.90	2.92
PAI-BOR Total T-score	10	50.80	10.64	9	75.11	7.61
PAI-BOR A Subscale T-score	10	54.00	12.73	10	72.00	2.45
PAI-BOR I Subscale T-score	10	49.90	9.89	10	69.50	8.40
PAI-BOR N Subscale T-score	10	52.10	8.65	9	73.89	10.75
PAI-BOR S Subscale T-score	10	45.30	8.33	9	64.33	12.05

Note: *MSI-BPD* McLean Screening Instrument for BPD, *PAI-BOR* Personality Assessment Inventory Borderline Scale, *A* affective instability subscale, *I* identity problems subscale, *N* negative relationships subscale, *S* self-harm subscale

**Table 2:** Independent Sample T-Tests

	t	df	Sig. (2-tailed)	Mean Difference^1^	Std. Error Difference	95% Confidence Interval of the Difference	
						Lower	Upper
Age in years	.75	18	.46	3.50	4.66	−6.30	13.30
MSI-BPD Total Score	3.87	14	.002	5.23	1.35	2.33	8.14
PAI-BOR Total T-score	5.67	17	.001	24.31	4.29	15.26	33.36
PAI-BOR A Subscale T-score	4.39	9.67	.001	18.00	4.10	8.82	27.18
PAI-BOR I Subscale T-score	4.78	18	.001	19.60	4.10	10.98	28.22
PAI-BOR N Subscale T-score	4.89	17	.001	21.79	4.45	12.39	31.19
PAI-BOR S Subscale T-score	4.04	17	.001	19.03	4.71	9.10	28.97

Note: 1 = group high in borderline traits – group low in borderline traits, *MSI-BPD* McLean Screening Instrument for BPD, *PAI-BOR* Personality Assessment Inventory Borderline Scale, *A* affective instability subscale, *I* identity problems subscale, *N* negative relationships subscale, *S* self-harm subscale

### Qualitative results of the emotional sensitivity interview

Four broad, interrelated themes emerged from the qualitative analyses of the emotional sensitivity interview. These included an overall negative experience of ES, confusion about internal states, experiences of invalidation of emotional responses, and preoccupation with the origin of ES, in the group with elevated borderline traits. In addition, differences in the process by which individuals talked about ES emerged.

#### Overall negative experience of emotional sensitivity

All participants above clinical cut-off for BPD considered themselves to be emotionally sensitive since childhood. Each recognized both the benefits and problems caused by their ES, but 9 out of 10 wished to change it at least in part, recognizing that their sensitivity was problematic. Nine out of ten participants high in borderline features predominantly defined ES as heightened emotional reactivity (i.e. “more likely to feel [emotions]”) and a tendency to have emotional reactions to even low-impact or non-emotional stimuli (i.e. “affected by situations others wouldn’t be”; having a “heightened sense of things that can hurt my feelings”); that is, they were often emotional (i.e. “It’s the same spectrum of emotions that everybody else experiences, but they experience them even more frequently”), quick to become emotional (i.e. “I’m very quick to snap on certain things”), felt a range of emotions (i.e. “I feel a lot of the emotional scale”) and felt these emotions intensely (i.e. “I feel deeper than other people do”, “all-consuming emotions”). The remaining participant defined ES as a mixed or balanced experience; that is, as heightened reactivity and a tendency to respond emotionally to their surroundings, but also as the improved ability to understand the emotions of others.

Comparably, participants with lower levels of borderline features considered themselves to be emotionally sensitive, but did not phrase this in as certain terms. For instance, 3 participants considered themselves to be only somewhat emotionally sensitive and 2 were decidedly “not” emotionally sensitive, although each was able to reflect upon certain situations in childhood and/or adulthood in which they were emotionally sensitive. Of the 8, at least partially emotionally sensitive participants, 7 recognized both the difficulties and benefits of their sensitivity but only 4 wanted to change it. Finally, only 6 out of 10 participants with lower levels of borderline features defined ES as heightened reactivity (i.e. “how easy someone can become emotional”, “how one easily reacts in different environments”) or a tendency to respond emotionally to stimuli (i.e. “being overly sensitive”, “how prone someone is to reacting emotionally to things…around them”). Three presented a mixed or balanced definition, while the remaining participant defined ES solely as the improved ability to understand and relate to others (Table 3).

**Table 3:** Emotional Sensitivity Interview Results

	Low borderline trait group		High borderline trait group			
	Yes	No	Somewhat	Yes	No	Somewhat
Emotionally Sensitive	5	2	3	10	0	0
Wish to Change Emotional Sensitivity	4	4	NA	9	1	NA

#### Reflection on internal states

Individuals with high levels of borderline pathology expressed a lack of understanding and confusion regarding multiple facets of their emotional lives. These individuals stated that they often did not know what they were feeling or why. One participant explained how as a child she would cry to express herself, even when not sad. When asked how ES has caused problems for her, one participant said, “well just confusion. You know, when is a good time to cry? When is a good time not to cry? ...I don’t want to be crying all the time; it’s just a good – just confusion all the time. I was confused and still is…if I’m not crying what are the other emotions”. Another participant could “understand people’s emotions but… [not] recognize it in themselves”. In comparison, individuals with low levels of borderline pathology who identified as being emotionally sensitive often defined ES as the ability to understand and reflect upon their emotional lives and the lives of others. One participant said ES was “being self-aware of how I’m feeling at any given time [and to be aware of] why people are feeling that way and why I’m feeling a certain way”. Another echoed those sentiments, stating it’s an “awareness of yourself and your feelings and the feelings of the people around you”.

Participants with high levels of borderline traits described how confusion over internal states resulted in feelings of alienation. One participant described “feeling like an outsider” from her own family. Their ES often distanced these individual from others or made them feel as though they stood apart from other people. When asked how being emotionally sensitive was for her, one participant said, “I didn’t understand why I was, why I felt things so strongly when other people weren’t”.

#### Perception of reactions to emotional experiences by close others

A third major theme that emerged was a perception or experience of invalidation of emotional experiences by close others, reported by participants with elevated borderline traits. When considering her family’s reactions to her ES as a child, one participant stated “I believe that they invalidated my sensitivity level”. This invalidation stemmed from her parents inability to understand her extreme emotional reactions. Subsequently, she expressed her confusion – she did not understand why they failed to see her perspective. One participant recounted a traumatic story from her childhood where she was “scared to death… [and her] siblings were like, meh, here we go again”. Upon considering her families reaction to the scenario she stated, “I don’t understand, I just don’t understand. At all…it was really weird...it’s like, you know, I don’t understand what’s going on”. Another participant recounted how as a child, his “hardest problem was whenever someone asked [him] why [he] was angry... [he] really didn’t know why [he] was angry” and felt that others required a reason for his emotional reaction, when he did not have one. Another participant recounted how her family would call her stupid because she “didn’t know what emotion to do”, distancing her from “normal” family members.

Like with confusion about internal states, individuals recounted how this perception of invalidation would lead to feelings of alienation. For example, when asked how ES “felt” for them, one participant said “you’re kinda sitting there thinking to yourself, why am I feeling this, when other people wouldn’t feel this bad”. For another, his emotional differences lead others to the conclusion that he was immature, incapable or un-intelligent – setting him apart from others.

In stark contrast, participants with low levels of borderline features reported supportive, validating responses to many of their emotionally sensitive experiences. At the least, they did not report feelings of alienation after rejection or invalidation. For example, one participant reported that although as a child she felt she was oftentimes not “heard” by her family and this made her feel lonely, she was able to connect with other emotionally sensitive people. As an adult, her family and friends reactions to her ES was “very relieving, definitely. The opposite of isolation, the opposite of not being understood”. Another participant stated that as a child her family was “fantastic” and “very supportive” when her ES would “fluctuate”. As an adult, when those around her suggested that she should or should not be emotionally sensitive, she said sometimes she does not agree with them, but it’s their right to think so – demonstrating an ability to separate the reactions of others from the validity of her own feelings. One final example comes from a participant who said that when they struggled emotionally in their youth, their parents would want them to learn to cope with their emotions better and would explain why the emotions they were feeling at the time (i.e. sadness after a breakup) would dissipate and how they would soon “get over it”. The participant felt their parents “reacted very appropriately and they never said or did anything that discouraged me from um letting them know how I felt in other instances”. Even when participants felt their emotions were ignored, criticized or responded to by close others with “you’re dumb”, “suck it up” and “stop crying”, individuals with low levels of borderline traits did not report subsequent feelings of invalidation or alienation and still felt they benefited from their ES overall.

#### Consideration of the origin of emotional sensitivity

Many participants with high levels of borderline pathology appeared to be preoccupied with their early family life and how that related to their tendencies to be emotionally sensitive. When asked how their families reacted to their ES growing up, participants explained how: they lived in a “rocky household”, with parents in a violent marriage who were emotionally “unavailable”; they were given up for adoption and suffered physical, sexual and verbal abuse; their mother had multiple divorces which “played a role in [their] emotional sensitivity”; their parents both “had BPD traits”, were religious, strict and being disowned was what they believe “eventually took [my] um, being over-overly emotional into um, a disorder of, of BPD”. When asked if she was always emotionally sensitive one participant said “yes. Absolutely” and that she believed her ES was because “from the time she was conceived” her mom was anxious and depressed during the pregnancy, affecting her development.

The origin of ES was rarely considered by those with low levels of borderline traits. Many participants suggested their ES (sensitivity/awareness of their emotions and the emotions of others) developed over time - either through interactions with friends, family and the mental health care field or with age. Another suggested ES was simply an inherent trait that would be “hard to develop”.

#### Process

In general, interviews with individuals high in borderline pathology were difficult to follow and can be described as generally low on coherence in terms of their ability to sequence thoughts, convey meaning and respond to the question at hand. The following example was a response to the question “have you always been emotionally sensitive?”: “uh anybody who knows me would tell you that. From the time that I was conceived, I believe, uh, I think that’s got, you know, something to do with it cuz my mother was really anxious and depressed when she was pregnant with me and she cried all the time and was so tense that her arm went numb because her muscles were so tense that she had to get a shot so I’ve heard those stories. I know I came out colicky. I know I had baby sitters refuse to baby sit for me, even from a very, very from an infant age, infancy. And, you know, I was just kicking and screaming from the time I came out”.

When asked how it feels to be emotionally sensitive, one participant said, “when I went to Kindergarten, or pre-Kindergarten, and children uh, you know, would make fun of my lunch box or my lunch box all of a sudden would have you know like, what seemed like hundreds of ants and nobody else’s did, it was, it, it was just ridiculous. I couldn’t, you know, screen this horrible pain inside, of embarrassment, of just [one word, garbled], oh my god, my mother doesn’t love me because the ants”. When asked how their family reacted to their ES as children, a participant instead described their *parents’* ES and insensitivity. They went into detail regarding their fathers’ struggle with depression and their mothers’ lack of understanding towards this. They also briefly mention how their parent’s behavior affected their life, however they did not describe how their family reacted to their own ES.

Interviews with participants low in borderline pathology were generally more coherent. Participants largely responded directly and concisely to interview questions, although at times had little to say regarding the negative impact or complications that ES played in their lives. Additionally, even when speaking about emotional distress, no participants with low levels of borderline pathology became emotionally dysregulated. In comparison, the audio of individuals with high levels of borderline pathology often reflected distress in their voices and 2 participants cried during the interview.

## Discussion

Emotional sensitivity is a construct of academic interest [2, 3, 6] and clinical utility [1] in borderline personaltiy disorder. However, despite its apparent significance in the conceptualization of BPD etiology, the definition and operationalization of ES remains nebulous [10]. Additionally, work in this area has not decisively indicated that significant differences between borderline and healthy populations even exist. These deficits in our understanding of ES may be due to a lack of a standardized definition and measurement technique or an insufficient understanding of the experience of ES. Against this background, the aim of the current study was to explore the meaning and experience of ES using qualitative methodology in individuals who self-identify as emotionally sensitive. We recruited individuals who identify as being emotionally sensitive from community samples to contribute to the nomological net [23], supporting a construct highly relevant to theories of borderline personality pathology.

Qualitative results of the ES interview suggest that ES, for those identifying themselves as emotionally sensitive, is a heightened emotional reactivity to stimuli, including the emotions of other individuals, [6] or a tendency to have emotional reactions to even low impact stimuli [8]. This finding aligns with the biosocial model’s conceptualization of ES in BPD [2, 3], but it is important to emphasize that this definition of ES was partially endorsed by *both* individuals with high and low levels of borderline features in the current study. While the defnition of ES largely appeared to be indistinct across groups, the *response to* being emotionally sensitive and the reported effect ES has on individuals appears to distinguish those with high and low levels of borderline features. In the group with high borderline traits, more participants defined their ES as being completely negative, and although they recognized the benefits of ES, they still wished to reduce it. Emotional sensitivity interviews of the group with high levels of borderline features also conveyed confusion and lack of understanding of emotions, a lack of narrative coherence and a preoccupation with etiological themes.

It is important to note that in the current study ES was not measured quantitatively and it is therefore unclear whether the groups significantly differed in levels of ES. Higher overall levels of ES in the group with high borderline features may account for the greater feelings of distress and more negative perceptions of ES observed in said group. Given that the majority of participants in both groups identified as being ES, however, it is also possible that it is not the trait of ES per se which is maladaptive, but rather the *meaning* an individual attaches to the trait that may be problematic. Differently, it is not the trait itself that associates with maladaptiveness, but potential social-cognitive mechanisms through which ES may cause distress. As described by Linehan [3], when an emotionally sensitive individual is repeatedly invalidated by their environment, they may experience their ES as inherently bad, wrong, different and shameful. This can cause feelings of distress via cascading alienation. These individuals feel something is “wrong” with them, alienating them from “normal” people. When close others do not understand them or their ES, this reinforces their belief that they are indeed different from others, resulting in further feelings of alienation and withdrawal. Therefore, participants with elevated borderline traits may have reported much more feelings of distress from their ES due to a subjective experience of alienation from others, something not seen in the group without elevated borderline traits. This suggests that ES is not a maladaptive characteristic of an individual, but a “mismatch” of ES levels with the environment, in addition to a failure to make sense of ES in that environment, but also in relation to the self. In sum, individuals with high levels of borderline pathology know they are different from others and have sought validation of their ES from others. When their emotional experiences are not validated, feelings of alienation are common and can become part of a cascading cycle with ES, where a perceived lack of understanding by others and subsequent feelings of alienation lead to increased ES, and so on.

Although the relations between invalidation, shame, self-invalidation and emotional sensitivity are delineated in etiological models of BPD [2, 3] to our knowledge, this may be the first study to empirically describe these phenomena from the patient perspective. Results of the current study suggest that the meaning of ES does not qualitatively differ depending on your level of borderline features. However, those with greater borderline features appear to respond to their ES in a different way. They are preoccupied with the etiology of their ES, attribute its development to their early family life, have difficulty accepting their ES and often wish they could reduce it. Thus, while we have long known of emotion dysregulation, shame and invalidation in BPD, this study highlights how social cognitive mechanisms may impact one's level of distress and response to being ES.

### Limitations and future directions

Despite the current study’s contribution to the understanding of ES in individuals with high and low levels of borderline traits, it has some limitations. The current study was qualitative, and therefore sought to advance our understanding of the construct of ES from the patient perspective. Even so, the sample size was small, and study results related to group differences in particular should be seen as preliminary.

A second limitation is the study’s qualitative nature itself. As an investigation into the subjective experience of ES, the current study in unable to quantitatively contribute to measurement of the construct. For example, it is not entirely clear that participants conceptualized emotional sensitivity *only* as sensitivity to stimuli. A few participants included in their definition or discussion feelings of greater emotional intensity than others – the second “step” in models of emotion dysregulation. This suggests the current study may not purely capture the unique experience of ES but of other components in the emotion dysregulation process as well. However, the majority of participants did define ES as, and focus their discussion on, sensitivity to environmental and interpersonal stimuli. This distinction between emotional sensitivity and intensity may be an area in need of future consideration given that some measures of emotion dysregulation have suggested through factor analysis that these constructs may not be distinct in measurement [11]. Future studies should complement the qualitative approach used here with quantitative self-report measures or experimental measures in a larger sample to clarify these distinctions. It is only through multiple levels of analyses that the nomological network supporting this construct can be fully determined [23].

The evaluation of substantive relations between the construct of ES and constructs that emerged in this study would also be of value. For example, it may be worthwhile to simultaneously investigate the relations between ES, feelings of interpersonal alienation or integration, shame or self-judgment and borderline features or psychological distress. It is possible that two individuals with similar elevated indications of ES show differing levels of distress, mediated by feelings of alienation. Using longitudinal approaches, such as experience sampling methodology, the “real-time” relation between these constructs may be clarified. Experimental approaches which induce feelings of alienation through exclusion may also be significant for clarifying the relation between ES and subsequent distress.

There are also limitations to the current study’s design. Participants did not complete a previously developed measure containing ES such as the Emotion Dysregulation Measure or the Emotion Reactivity Scale [11, 12]. Therefore, it is unclear whether individuals with high and low levels of borderline features quantitatively differed in self-reports of ES. In the current study, we speculated that differential levels of distress, across groups, in response to ES may indicate the role of social-cognitive mechanisms in this relation. However, as we did not quantitatively measure ES, it is unclear whether the group high in borderline features simply had greater “levels” of ES which contributed to their greater feelings of distress. Additionally, the current study did not include a clinical comparison group. It is possible that negative experiences of ES are not unique to BPD and may be generalizable to other forms of psychopathology. Finally, the qualitative methodology utilized in the current study did not allow for calculation of inter-rater reliability between interview coders. Although best-practices were followed in order to ensure validity and reliability of results of the current study, future work in this area may make use of qualitative approaches which do allow for calculation of inter-rater reliability indices.

A final limitation is the study’s recruitment strategy and subsequent sample. Most participants in the group with high borderline traits were individuals who responded to the ad posted on the blog of the DBT center director. Although not necessarily DBT patients, these individuals may have had increased exposure to terminology and constructs found in DBT. It is possible this influenced their interview responses, however as mentioned, not all participants in the borderline group were recruited via this ad nor were they DBT patients. Additionally, 2 participants did not consider themselves to be ES. This may have affected results, however both participants were in the group with lower levels of borderline features, where expectations of ES were also lower. Additionally, they were both able to define and reflect upon times when they were emotionally sensitive.

## Conclusions

Notwithstanding the above limitations, the current study contributes to our understanding of the construct of ES through the eyes of those identifying themselves as emotionally sensitive. Those high in borderline traits exhibited feelings of distress, alienation and isolation as a result of their ES. Additionally, themes of familial invalidation permeated the narratives of the group high in borderline features. These results highlight the social cognitive mechanisms through which ES-related distress may be brought about.

## Abbreviations

BPD: Borderline personality disorder
DBT: Dialectical behavior therapy
ES: Emotional sensitivity
fMRI: Functional magnetic resonance imaging
MSI-BPD: McLean Screening Instrument for BPD
PAI-BOR: Personality Assessment Inventory – Borderline Features Scale
